# 

**Published:** 1888-05

**Authors:** 


					•v6l viii.
MAY, 1888.
No. &
THE
HOMEOPATHIC pHY^iClAjI,
A Monthly Journal of Homoeopathic Materia Medica 
and Clinical Medicine.
“ Be is a freeman whom trulht makes free, ami all are slaves beside”
“Seek the Trufh:. come whence it may, cost what it will”
KDITEDAND PUBLISHED BY
Edmund j. lee, m. D.,
AND
WALTER M. JAMES, M. D.
PHILADELPHIA:
No. XXS3 SZPIRTTCK STREET.
X. O 3ST X) O TT s
ALFRED HEATH & CO., Sole Agents fob Great Britain, 
114 Ebury Street,- Eaton Square.
^B~Yearly Subscription, $2.50, invariably in advance. Single numbers, 25 etc.
Entered at the Philadelphia Poet-Office as Second-Class Mail Matter.
				

